# An Adeno-Associated Virus-Based Intracellular Sensor of Pathological Nuclear Factor-κB Activation for Disease-Inducible Gene Transfer

**DOI:** 10.1371/journal.pone.0053156

**Published:** 2013-01-03

**Authors:** Abdelwahed Chtarto, Olivier Bockstael, Elias Gebara, Katia Vermoesen, Catherine Melas, Catherine Pythoud, Marc Levivier, Olivier De Witte, Ruth Luthi-Carter, Ralph Clinkers, Liliane Tenenbaum

**Affiliations:** 1 Laboratory of Experimental Neurosurgery, Université Libre de Bruxelles (ULB), Brussels, Belgium; 2 Faculty of Medecine, Institut de Recherche Interdisciplinaire en Biologie Humaine et Moléculaire (I.R.I.B.H.M.), Université Libre de Bruxelles (ULB), Brussels, Belgium; 3 Brain and Mind Institute, Ecole Polytechnique Fédérale de Lausanne (EPFL), Lausanne, Switzerland; 4 Department of Pharmaceutical Chemistry and Drug Analysis, Center for Neurosciences (C4F), Faculty of Medecine and Pharmacy, Vrije Universiteit Brussel, Brussels, Belgium; 5 Department of Clinical Neuroscience, Lausanne University Hospital, Lausanne, Switzerland; Ospedale Pediatrico Bambino Ges?, Italy

## Abstract

Stimulation of resident cells by NF-κB activating cytokines is a central element of inflammatory and degenerative disorders of the central nervous system (CNS). This disease-mediated NF-κB activation could be used to drive transgene expression selectively in affected cells, using adeno-associated virus (AAV)-mediated gene transfer. We have constructed a series of AAV vectors expressing GFP under the control of different promoters including NF-κB -responsive elements. As an initial screen, the vectors were tested *in vitro* in HEK-293T cells treated with TNF-α. The best profile of GFP induction was obtained with a promoter containing two blocks of four NF-κB -responsive sequences from the human JCV neurotropic polyoma virus promoter, fused to a new tight minimal CMV promoter, optimally distant from each other. A therapeutical gene, glial cell line-derived neurotrophic factor (GDNF) cDNA under the control of serotype 1-encapsidated NF-κB -responsive AAV vector (AAV-NF) was protective in senescent cultures of mouse cortical neurons. AAV-NF was then evaluated *in vivo* in the kainic acid (KA)-induced status epilepticus rat model for temporal lobe epilepsy, a major neurological disorder with a central pathophysiological role for NF-κB activation. We demonstrate that AAV-NF, injected in the hippocampus, responded to disease induction by mediating GFP expression, preferentially in CA1 and CA3 neurons and astrocytes, specifically in regions where inflammatory markers were also induced. Altogether, these data demonstrate the feasibility to use disease-activated transcription factor-responsive elements in order to drive transgene expression specifically in affected cells in inflammatory CNS disorders using AAV-mediated gene transfer.

## Introduction

The clinical implementation of drug-dependent regulatable viral vectors for gene therapy has been hampered by the adverse effects of the pharmacologic inducer and the immunogenicity of the artificial transactivator generally containing viral and/or bacterial elements [1], [2]. Ideally, gene therapy for CNS diseases should be modulated according to the severity and the evolution of the disease. In addition, the transgene should be specifically expressed in the affected brain region and cell types. As an alternative to drug-mediated regulatory systems, we considered the possibility that regulatory DNA sequences binding transcription factors that are activated in pathological conditions could be used to generate pathology-inducible promoters responding to specific endogenous stimuli [3]–[5].

The NF-κB family of transcription factors consists of 5 members, defined by their Rel homology region, forming homo- and heterodimer complexes [6].

While inactive, NK-κB dimers are bound to IκB proteins and retained in the cytoplasm. During activation, IκB is degraded and the NF-κB transcription factors are released and translocate to the nucleus where they regulate the transcription of numerous genes [7]. Stimulation of the NF-κB pathway is a key pathologic component of numerous CNS disorders [8]. In particular, NFκB activation is a major pathogenic component in the kainic acid (KA)-induced rat model of status epilepticus (SE) [9], [10], one of the most widely used and best characterized animal models for temporal lobe epilepsy (TLE) [11]–[14]. This model displays characteristic neuropathological aspects of the disease including hippocampal sclerosis (neuron loss and gliosis), neuroinflammation, synaptic reorganization, such as mossy fibre sprouting, and the chronic recurrence of spontaneous seizures [15]–[19].

NFκB activation resulting from increases in inflammatory cytokines has also been involved in pathological processes related to aging [20] and Alzheimer Disease [21]. Additional data suggest that NFκB activation is not an autocompensatory response, since NFκB activation fails to protect neurons against apoptosis associated with long-term culturing or dual TNFα and amyloid-beta toxicity [22].

Harnessing disease-mediated NF-κB activation to drive transgene expression in CNS cells could constitute an interesting intracellular approach to anti-inflammatory intervention [23].

Several lines of transgenic reporter mice using tandem NFκB-responsive promoter sequences have exhibited heterogeneous phenotypes. This can likely be explained by the fact that the Rel family of transcription factors includes numerous variants whose abundance varies depending on the tissue and which bind with differential affinities to different variants of the NFκB response element (NFκB-RE). We therefore reviewed these data carefully prior to selecting NFκB-REs for testing in our regulatable gene therapy vectors. Transgenic mice using the NFκB responsive sequence from the Igκ light chain promoter mainly express the reporter gene in immune organs and intestine and transgene expression is further inducible by TNFα, IL1β or LPS in other organs such as lungs and liver [24]. In contrast, the NFκB responsive element present in the HIV promoter is expressed in immune organs, but is also constitutively active in the CNS [25]. The regulatory region of human neurotropic polyoma virus JCV which contains a NFκB-RE variant that differs from the HIV sequence by only one nucleotide [26] is active in infected astrocytes and oligodendrocytes presumably due to TNFα stimulation of these glial cells [27].

A neuroinflammation-responsive AAV vector based on JCV NFκB-RE consensus transcriptional sequences was designed. We first tested this NFκB-inducible vector in an *in vitro* model of brain aging [28] and showed that its delivery of glial cell line-derived neurotrophic factor (GDNF) cDNA under the control of the NFκB-inducible promoter enhanced survival of aging cortical neurons in culture. We subsequently showed that hippocampally-delivered, the NFκB-inducible AAV vector responded to systemic KA injection in neurons and astrocytes in specific subregions of the CA1, CA3 hippocampal layers and stratum orens where inflammatory markers were also induced. Altogether, these results bode positively for the utilization of our novel pathology-inducible vector design for anti-inflammatory gene transfer applications.

## Materials and Methods

### Plasmids

The pNFκB-d2EGFP plasmid containing 4 NFκB responsive element (NFκBRE) fused to a minimal thymidine kinase promoter (mTK) was purchased from Clontech (Palo Alto, CA, USA) The pHpaI-EGFP self-complementary AAV vector was a kind gift from D.McCarty and RJ Samulski [29].

**Figure 1:**
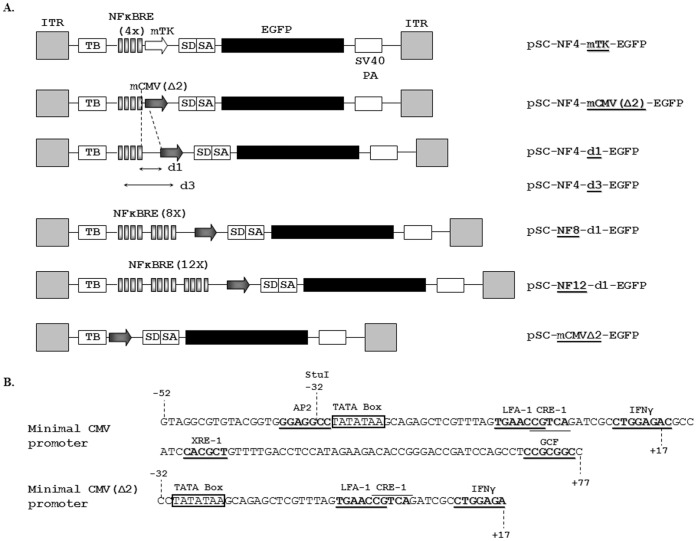
Description of the NF-κB-responsive AAV vectors. A. Schematic diagram of NF-κB-responsive AAV vectors used in this study. ITR: AAV Inverted Terminal Repeat; TB: Transcriptional Blocker; NFκBRE (4X): four copies of the JC virus NFκB consensus sequence; mTK: herpes simplex virus minimal thymidine kinase promoter; SD/SA: Splice Donor/Splice Acceptor sequence [72]; mCMVΔ2: improved minimal CMV promoter; d1, d3: distance separating the last NFκB consensus sequence and the minimal promoter, respectively, 31 and 71 bp. B. Sequence analysis of the transcription factor binding sites within the minimal CMV and the minimal CMV Δ2 promoters. The mCMV promoter originates from position −52 to position +77 in the wild-type promoter [45]. Analysis of the mCMV sequences highlighted the presence of several transcription factor binding sites : AP2, LFA-1, CRE-1, IFNγ, XRE-1 and GCF. The mCMVΔ2 was obtained by deleting the sequence upstream of the TATA box (−52 to −32) and the sequence downstream of the IFNγ site (+17 to +77) resulting in the removal of AP2, XRE-1 and GCF sites.

We first constructed pSC-NF4-mTK-d2EGFP by replacing the EcoRI-SalI fragment of pHpaI-EGFP containing the CMV promoter, EGFP coding sequence and SV40 polyA with a NotI-SalI fragment from pNFκB-d2EGFP containing a transcription blocker site, a composite NFκB-responsive promoter (4 copies of the NFκB-responsive element fused to a minimal thymidine kinase promoter), destabilized GFP (d2EGFP) and SV40 polyA. Into the obtained vector, d2EGFP was replaced with EGFP to generate pSC-NF4-mTK-EGFP (Fig. 1A). The pSC-mCMVΔ2-EGFP was derived by replacing the mTK promoter in pSC-NF4-mTK-EGFP with a new modified minimal CMV promoter (mCMV) named mCMVΔ2 in which sequences 5′and 3′to the TATA box were deleted. To obtain pSC-NF4-d1-EGFP and pSC-NF4-d3-EGFP we increased the distance between the NFκBRE and the mCMVΔ2 promoter by introducing stuffer sequences of various lengths. The pSC-NF-Ctrl was obtained by excising a fragment containing NFκBRE in pSC-NF4-mCMVΔ2-EGFP. To construct pSC-NF8-d1-EGFP and pSC-NF12-EGFP, we replaced the NF4 in pSC-NF4-mCMVΔ2-EGFP respectively by two or three blocks of 4 NFκBRE, separated by 16 bp. The pSC-NF8-d1-GDNF vector was obtained by replacing the EGFP coding sequence in pSC-NF8-d1-EGFP by the human GDNF cDNA (a kind gift from Dr Nicole Deglon, Lausanne Switzerland).

### Cell Line

The HEK-293T cell line was purchased from Q-One Biotech (Glasgow, UK) and cultured in Dulbecco’s modified Eagle’s medium (DMEM) supplemented with 10% FCS (Gibco BRL, Life Technologies, Merelbeke, Belgium).

### Transfections

For the analysis of the inducibility of the vectors, HEK-293T cells were transfected using the calcium phosphate coprecipitation method. Fifty-thousand cells in 6-wells were transfected with 250 ng DNA. Forty-eight hours later and 5 hours before analysis, transfected cells were treated or not treated with TNFα (Invitrogen) (100 ng/ml). Cells transfected with AAV-NF-EGFP were analyzed for GFP expression on a FACStar analyser/sorter (Becton Dickinson). To analyse the inducibility of AAV-NF-hGDNF vectors in HEK 293T, the supernatants were harvested 4 h after changing the medium to measure GDNF concentration using a commercial ELISA assay (Human GDNF CytoSets, catalog #CHC2423, BioSource, Nivelles, Belgium). The transfection efficiency was normalized using a plasmid constitutively expressing GFP protein.

### Viral Production

To produce recombinant AAV2/1 viral stocks, HEK-293T cells (5.0×10^6^ cells seeded on 10 cm plates) were cotransfected, in a 1∶1 molar ratio, with the vector plasmid (3 µg/plate) together with the helper/packaging plasmid pD1rs (10 µg/plate) expressing the AAV viral genes (rep gene from AAV serotype 2 and cap gene from AAV serotype 1) and the adenoviral genes required for AAV replication and encapsidation (Plasmid Factory, Heidelberg). Fifty hours post-transfection, the medium was discarded and the cells were harvested by low-speed centrifugation and resuspended in Tris pH 8.0, NaCl 0.1 M. After three cycles of freezing/thawing, the lysate was clarified by 30 min centrifugation at 10 000 *g*, treated with DNase at 37°C for 30 min, and centrifuged at 10 000 *g* for 30 min to eliminate the residual debris. The virus was further purified by iodixanol gradient followed by QXL-sepharose chromatography, according to a well-established method [30]. Viral genomes (vg) were titrated by quantitative PCR as previously described [31]. Titers were 1.95×10^10^ and 7.3×10^11^ vg/ml respectively for rAAV1-NF8-d1-EGFP and rAAV1-NF8-d1-hGDNF.

### Cultures and Infection of Cortical Neurons

Neuronal cultures were prepared from embryonic day 16 Sprague-Dawley rat fetuses using methods similar to those described previously [32].

Neurons were plated in 96-well or 48-well dishes (CostarTM) previously coated with poly-L-lysine (MW 30,000–70,000).

Half of the medium was replaced, weekly, with freshly prepared NeurobasalTM medium (Invitrogen) supplemented with 2% B27 (Invitrogen), 1× penicillin-streptomycin, 0,5 mM L-glutamine, and 15 mM KCl. On *in vitro* Day 3, cells were infected at a multiplicity of infection of 10^4 ^vg/cell.

### Immunocytochemistry

Primary cultures were fixed with 4% paraformaldehyde (PFA) for 20 min at 4°C. Cultures were washed 3×10 min with PBS and then incubated for 1 h in a blocking solution of PBS supplemented with 10% Bovine Serum Albumin (PAA laboratories GmbH, Austria) and 0.1% Triton in PBS. The cells were then incubated overnight at 4°C in a blocking solution containing mouse monoclonal anti-NeuN antibody (1∶400, Chemicon), goat polyclonal anti-PSD95 (1∶500, Abcam).

Cells were washed 3×10 min with PBS and then incubated for 1 h with a fluorescent secondary Cy3-conjugated goat anti-mouse antibody (1∶1000, Jackson ImmunoResearch Laboratories), Alexa 660 donkey anti-goat antibody (1∶500, Invitrogen) followed by 3×10 min PBS washes.

Images of immunostained cells were acquired with a BD pathway 435 Openaccess instrument for cell counting. Image analysis was performed with ImageJ software (US NIH).

For activated NFκB staining, the same protocol was applied using a mouse monoclonal IgG3 anti-activated NFκB recognizing an epitope overlapping the nuclear location signal of the p65 subunit of the NFκB heterodimer thus selectively binding to the activated form of NFκB [33] (Millipore, catalog # MAB3026). The antibody was diluted 1∶100 as primary antibody and a biotin-conjugated donkey anti-mouse IgG (Jackson ImmunoResearch, cat number: 715-065-150) diluted 1∶500 was used as secondary antibody. For nuclear counterstainings, cells were incubated in the Hoescht 33258 dye (Sigma-Aldrich) diluted at 1 µg/ml in TBS for 15 min. Three 10 min washes in TBS of were performed between each step. Images of immunostained cells were obtained by confocal microscopy (Lasersharp version 3.2 (Biorad, Hercules, CA) coupled to Axiovert 100 microscope, (Carl Zeiss, Gottingen, Germany)). The intensity of the staining of individual nuclei was quantified by measuring optical densities using the Image J software (NIH, USA).

### Glial Cell Line-derived Neurotrophic Factor (GDNF) Determination by ELISA

Medium was harvested from rAAV1-NF8-d1-EGFP- and rAAV1-NF8-d1-hGDNF-infected cortical neuron cultures at the indicated time points.GDNF concentrations were measured using a commercial ELISA assay (Human GDNF CytoSets, catalog #CHC2423, BioSource, Nivelles, Belgium) and expressed in pg/ml. Recombinant human GDNF (provided by the manufacturer) was used to establish the standard curve.

### Kainic Acid-induced Post-status Epilepticus Ral Model of Temporal Lobe Epilepsy

#### Intracerebral AAV vector injections

Adult male Wistar rats (Charles River) weighing approx. 200 g were housed and treated according to the Belgian law. The protocols were in accordance with national rules on animal experiments and were approved by the Ethics Committee of the Faculty of Medicine of the “Université Libre de Bruxelles”. Animals were anesthetized with a mixture of ketamine (Imalgène 1000, Merial; 100 mg/kg) and xylazin (Rompun, Bayer; 10 mg/kg) and placed in a Kopf stereotaxic frame (Kopf Instruments, Tujunga, CA, USA). The injection coordinates in the hippocampus were 4.8 mm posterior, 4.6 mm lateral to bregma, and 4.4 mm below the dural surface and in the cerebellum: 11.96 mm posterior, 2.0 mm lateral to bregma, and 2.5 mm below the dural surface. The injection rate was 0.2 µl/min. The needle was left in place for 1 min before a slow withdrawal over an additional minute.

#### Intraperitoneal kainic acid injections

The induction of status epilepticus was performed as described earlier [34], [35] **.** Briefly, consecutive intraperitoneal kainic acid (KA) injections (5 mg/kg, diluted in PBS, Nanocs®) were administered with a one hour interval. If a rat was nearing SE, half-doses (2.5 mg/kg) were given in order to reduce mortality. Control rats were injected with saline (NaCl 0.9%). Based on previous studies in which these animals were chronically monitored with video-electrocorticographical recordings, nearly all rats develop spontaneous seizures (99.95%) with the first unprovoked seizure recorded 11.68±6.86 days post-status epilepticus (n = 35).

This is in line with other groups applying the same status epilepticus induction procedure with individually-dosed kainic acid [16], [36].

Briefly, consecutive intraperitoneal kainic acid (KA) injections (5 mg/kg, diluted in PBS, Nanocs®) were administered with a one hour interval. If a rat was nearing SE, half-doses (2.5 mg/kg) were given in order to reduce mortality. Control rats were injected with saline (NaCl 0.9%).

Animals were sacrificed one week after intraperitoneal KA injection and perfused intracardiacally first with saline, then with 4% paraformaldehyde (PF4). Brains were postfixed for 24 hours in PF4.

The experiment was repeated 3 times with similar results.

### Immunofluorescence on Brain Sections

#### GFP labeling

Coronal brain sections (50 µm) obtained using a vibratome (Leica Microsystems, Wetzlar, Germany) were sequentially incubated in: i) THST (50 mM Tris, 0.5 M NaCl, 0.5% Triton X100 (Merck, Frankfurter, Germany) pH7.6) containing 10% horse serum for 2 hours; ii) polyclonal rabbit anti-GFP (1∶3000, Molecular Probes, Invitrogen, Carlsbad, CA) diluted in THST containing 5% horse serum for 16 hours at 4°C; iii) donkey anti rabbit IgG conjugated with biotin (Amersham, GE Healthcare, Munich, Germany) diluted 1∶600 in THST containing 5% horse serum, 2 hours at room temperature; iv) streptavidin conjugated to cyanine 2 (1∶300; Jackson ImmunoResearch, West Grove, PA) in THST containing 5% horse serum, 2 hours at room temperature. Three washings in TBS (Tris 10 mM, NaCl 0.9%, pH7.6) of 10 min. were performed between each step.

Sections were mounted using FluorSave mounting fluid for fluorescence (Calbiochem, Merck, Frankfurter, Germany) and photographed using a Zeiss Axiophot 2 microscope equipped with FITC and TRITC filters (Car Zeiss, Göttingen, Germany) as well as an AxioCam digital camera (Carl Zeiss, Gottingen, Germany). Images were acquired as jpeg files using the KS300 software (Car Zeiss, Gottingen, Germany).

The number of GFP-positive cells was evaluated by stereological procedures based on the Cavalieri principle (Sterio, 1984). For each animal, serial sections with an interval of 500 µm were analyzed by means of the optical fractionator of the Stereoinvestigator software (MBF Bioscience, Williston, VT) connected to the microscope with a CCD video camera (Leica Microsystems, Wetzlar, Germany).

#### GFP:NeuN and GFP:GFAP co-labelings

For double immunofluorescence, these incubations were combined with mouse monoclonal antibodies (anti-NeuN (1∶200, Chemicon, Millipore, Billerica, MA) or anti-glial fibrillary acid protein (GFAP, 1∶200, Chemicon, Millipore, Billerica, MA)) (step ii); and donkey anti-mouse IgG coupled to cyanine 3 (1∶200; Jackson ImmunoResearch, West Grove PA) in THST containing 5% horse serum) (step iv).

Sections were mounted using FluorSave mounting fluid for fluorescence (Calbiochem, Merck, Frankfurter, Germany).

Co-labeling analysis were performed by confocal microscopy on pictures taken on at least three different sections within the transduction zone using an automatic image analysis system (Lasersharp version 3.2 (Biorad, Hercules, CA) coupled to Axiovert 100 microscope, (Carl Zeiss, Gottingen, Germany)). Pictures were then processed and analysed with the Image J software (NIH, USA).

#### GFP:IbaI co-labeling

For GFP:IbaI double immunofluorescence, the above described GFP labeling was combined with goat anti-IbaI (Abcam, cat number: ab 5076) followed by a donkey anti-goat A.568 antibody (Molecular probes, cat number A-11057) diluted 1∶500.

#### GFP:Olig2 co-labeling

For GFP:Olig2 double immunofluorescence, a chicken monoclonal anti-GFP antibody (Abcam, Cambridge, UK) at a 1∶1000 dilution was combined with rabbit polyclonal anti-Olig2 IgG (diluted 1∶500; Chemicon/EMD Millipore).

In order to better visualize the structure of the tissue, cerebellar sections were incubated in the Hoescht 33258 dye (Sigma-Aldrich) diluted at 1 µg/ml in TBS for 30 min. Three washings in TBS of 10 min were performed between each step.

Sections were mounted using Glycergel Mounting Medium for fluorescence (Dako, Belgium, cat number: C0563).

### Confocal Microscopy

Co-labeling analysis was performed on pictures taken on at least three different sections using a LSM510 NLO multiphoton confocal microscope fitted on an Axiovert M200 inverted microscope equipped with C-Apochromat 40×/1.2 N.A. and 63×/1.2 N.A. water immersion objectives (Zeiss, Iena, Germany).

The 488 nm excitation wavelength of the Argon/2 laser, a main dichroic HFT 488 and a band-pass emission filter (BP500–550 nm) were used for selective detection of the green fluorochrome (Cy2, Alexa 488).

The 543 nm excitation wavelength of the HeNe1 laser, a main dichroic HFT 488/543/633 and a long-pass emission filter (BP565–615 nm) were used for selective detection of the red fluorochrome (Cy3).

The nuclear stain Hoechst was excited in multiphotonic mode at 760 nm with a Mai Tai tunable broad-band laser (Spectra-Physics, Darmstad, Germany) and detected using a main dichroic HFT KP650 and a band-pass emission filter (BP435–485 nm).

Optical sections, 2 microns thick, 512 by 512 pixels, were collected sequentially for each fluorochrome. Z-stacks with a focus step of 1 micron were collected.

The data-sets generated were merged and displayed with the Zeiss Zen 2009 software and exported in LSM image format.

Countings were performed with the ImageJ 1.46 a software (NIH, USA). Figures were prepared with Adobe Photoshop CS3 software.

### Chromogenic Immunohistochemistry on Brain Sections

For CD11b staining, vibratome coronal brain sections (50 µm) were sequentially incubated in: i) 3% H_2_O_2_ in TBS (Tris 10 mM, 0.9% NaCl, Ph 7.6) for 30 min.; ii) THST (50 mM Tris, 0.5 M NaCl, 0.5% Triton X100 pH 7.6) containing 10% horse serum for 1 hour; iii) mouse monoclonal anti-CD11b (1∶500, Serotec, MorphoSys, Dusseldorf, Germany) in THST containing 5% horse serum overnight à 4°C; (step iii), then goat anti-mouse IgG conjugated with HRP (Molecular Probes, Invitrogen, Carlsbad, CA, from TSA kit) diluted in Blocking Reagent provided with the TSA kit (Molecular Probes, Invitrogen, Carlsbad, CA) was added for 2 hours at room temperature (step iv); and v) diaminobenzidine (Vector, NTL Laboratories, Brussels, Belgium), according to the manufacturer’s protocol. Sections were photographed using a Zeiss Axiophot 2 (Carl Zeiss, Gottingen, Germany) microscope.

For activated NFκB staining, the same protocol was applied using a mouse monoclonal IgG3 anti-activated NFκB recognizing an epitope overlapping the nuclear location signal of the p65 subunit of the NFκB heterodimer thus selectively binding to the activated form of NFκB [33] (Millipore, catalog # MAB3026). The antibody was diluted 1∶100 as primary antibody (step iv) and a goat anti-mouse IgG conjugated with biotin diluted 1∶200 was used as secondary antibody (step v). Densitometric analysis of the stainings was performed using the image J software (NIH, US).

### Statistical Analysis

All the statistical analysis was performed using the GraphPad Software.

Results were expressed as mean ±SEM and statistical significance was evaluated with one-way ANOVA Newman-Keuls or student T-test. Differences were considered as significant when p<0.05. Correlation analysis was evaluated with Pearson’s correlation test.

## Results

### 1. Design and *in vitro* Evaluation of NFκB-inducible AAV Vectors

NFκB-inducible reporter cassettes were constructed by fusing a minimal promoter with several repeats of the NFκB responsive elements (NFκB–RE) from the non-coding regulatory region of JC virus [26] upstream to an EGFP reporter gene. The promoter-reporter cassettes were then introduced in a self-complementary AAV vector which allows a rapid onset of transgene expression [37], [38]. In order to avoid influence of the AAV ITR promoter/enhancer activity on the NFκB-RE-containing promoter [39], [40], a transcriptional blocker site [41] was placed between the left ITR and the test promoters and a bidirectional SV40 polyA was placed between the transgene cDNA and the right ITR [42] (see Fig. 1).

Two different minimal promoters fused to four repeats of the NFκB-RE were compared. These consisted of a minimal thymidine kinase promoter [43] and a minimal CMV promoter [42] from which other putative transcription factor binding sites upstream and downstream of the TATA box were deleted, retaining only 3 downstream consensus sequences (CRE-1, LFA-1 and IFNγ). This minimal promoter will be hereafter designated as mCMVΔ2 (see Fig. 1A and Fig. 1B). The pSC-NF-Ctrl-EGFP plasmid containing mCMVΔ2, TB and the SV40 polyA but devoid of NFκB-responsive sequences was used as a control.

**Figure 2:**
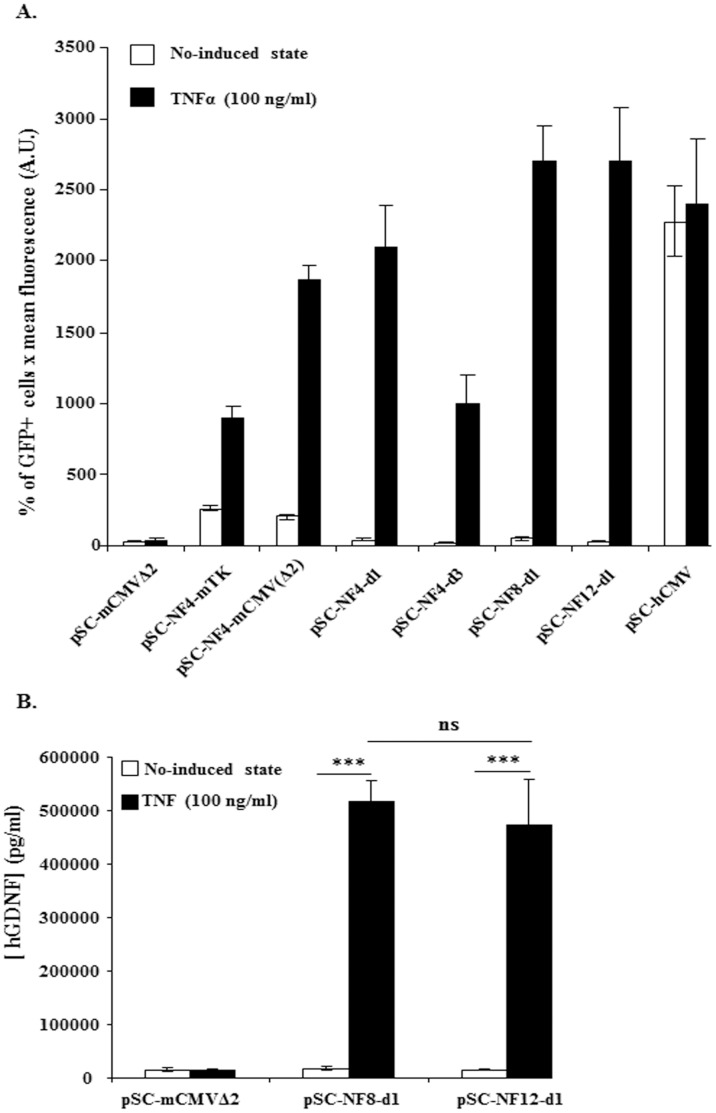
*In vitro* TNFα-mediated induction of NF-κB-responsive AAV vectors. HEK-293T cells were transfected with AAV-NF-EGFP (**A**) or with AAV-NF-hGDNF (**B**) vectors using the calcium phosphate coprecipitation method. 5×10^5^ cells in 6-wells were transfected with 250 ng DNA. Forty-eight hrs later, transfected cells were treated or not with TNFα (100 ng/ml) for 5 hrs. HEK-293T transfected with AAV-NF-EGFP were analyzed for GFP expression on a FACStar analyser/sorter (Becton Dickinson) (**A**). To analyse the inducibility of AAV-NF-hGDNF vectors in HEK-293T, the supernatant were harvested 4 h after changing the medium to measure secreted GDNF concentrations by a commercial ELISA assay (Human GDNF CytoSets, catalog #CHC2423, BioSource, Nivelles,Belgium) (**B**). For both AAV-NF8-d1-hGDNF and AAV-NF12-d1-hGDNF vectors, the differences between the TNFα-treated and untreated cultures were highly significant. The differences between the basal levels and the induced levels for AAV-NF8-d1-hGDNF versus AAV-NF12-d1-hGDNF are not significant (**B**). (***, p<0.001; ns, p>0,05, one-way ANOVA Newman-Keuls Multiple Comparison Test). Data are from one representative out of three experiments (3 separate transfections). Bars represent means ± standard deviations (SD) (**A**) or means ± standard error of the means (SEM) (**B**) from triplicate wells. A.U., arbitrary units.

The test vectors were transfected into HEK-293T cells, which were left untreated or exposed to TNFα (100 ng/ml). HEK-293T cells were chosen for these studies based on previous data suggesting that they have little or no NFκB activation under basal conditions [44]. Fig. 2A shows that the use of the mCMVΔ2 promoter resulted in a higher cytokine-induced level of GFP expression than the minimal TK promoter (p<0.001, one way ANOVA, Newman-Keuls multiple comparison Test) without increasing basal level and thus it was selected for the following constructions. As expected, the control (pSC-NF-CtrL-EGFP and pSC-NF-CtrL-hGDNF) vectors were not inducible by TNFα (p>0.05) (Fig. 2A and 2B).

#### i) Optimisation of the distance between NFκB-RE and mCMVΔ2

The distance between enhancers and the transcription initiation site is known to be important for the interaction between the proteins involved in the trancriptional complex [45]. In order to optimize the inducibility of the AAV-NF vector, we tested various spacings between the NF-κB repeats and the TATA box of the mCMVΔ2 promoter.

Increasing the distance from 6 bp (pSC-NF4-mCMVΔ2) to 31 bp (pSC-NF4-d1) or 71 bp (pSC-NF4-d3) resulted in significant (p<0.01) distance-dependent decreases of the basal reporter level. The profile was different for the induced levels however. Inducibility was maintained up to a distance of 31 bp (p>0.05, pSC-NF4-mCMVΔ2 versus pSC-NF4-d1) and decreased at a distance of 71 bp (p<0.001, pSC-NF4-mCMVΔ2 versus pSC-NF4-d3). On the basis that a spacing distance of 31 bps maintained high inducible expression levels while decreasing basal expression (compared to the shorter spacing of 6 bp) the 31 bp spacing incorporated in pSC-NF4-d1 was carried forward for further development.

#### ii) Optimisation of the number of NFκB-RE

Starting from pSC-NF4-d1 (with 4 NFκB-REs), the number of repeats was increased to 8 and 12 (corresponding to pSC-NF8-d1 and pSC-NF12-d1; see Fig. 1A). pSC-NF8- d1 and pSC-NF12-d1 showed basal levels of GFP expression similar to pSC-NF4-d1 whereas the TNFα-induced levels were higher than pSC-NF4-d1 for both plasmids (p<0.01, pSC-NF4-d1 versus pSC-NF8-d1 and pSC-NF4-d1 versus pSC-NF12-d1) (Fig. 2A). Using two reporter genes (EGFP and hGDNF), no significant difference in TNFα-induced levels was observed between pSC-NF8-d1 and pSC-NF12-d1 (Fig. 2A and 2B). Based on its retaining a larger residual cloning capacity (approx. 900 bp), pSC-NF8-d1 vector was selected for further testing.

### 2. Enhanced Survival of Senescent Cortical Neurons Mediated by NF-κB-inducible GDNF Expression

We then wanted to test whether an effect of a therapeutic transgene could be conveyed by the rAAV2-NF8-d1 recombinant virus. To address this question, the rAAV2-NF8-d1 vector expressing the human GDNF cDNA was evaluated in an *in vitro* model of brain aging.

To determine which capsid serotype would be suitable for our primary cells in culture, we evaluated control cultures infected with AAV vectors expressing EGFP under the control of the CMV promoter transencapisdated into serotype 1, 2 and 5 capsids (5,000 cells/well; 10^4^ vg/cell). This relatively low multiplicity of infection was chosen to avoid previously described AAV vectors-related toxicity in primary neurons cultures [46]. GFP-positive cells were observed from day 3 after infection and their detectable numbers reached 170 (rAAV2/1), 40 (rAAV2/2) and 92 (rAAV2/5) at 5 days post-infection (data not shown). Therefore, serotype 1 was selected for further experiments.

We then proceeded to test the efficacy of our new vector in long-term cultures of E16 cortical neurons and astrocytes in serum-free medium. In this model, neurons gradually mature and form synapses but eventually undergo apoptosis starting at approximately 35–40 days *in vitro.* 5,000 cells were infected with rAAV2/1-NF8-d1-EGFP (n = 10) as a control or rAAV2/1-NF8-d1-GNDF (n = 10) at a multiplicity of 10^4^ vg/cell.

**Figure 3:**
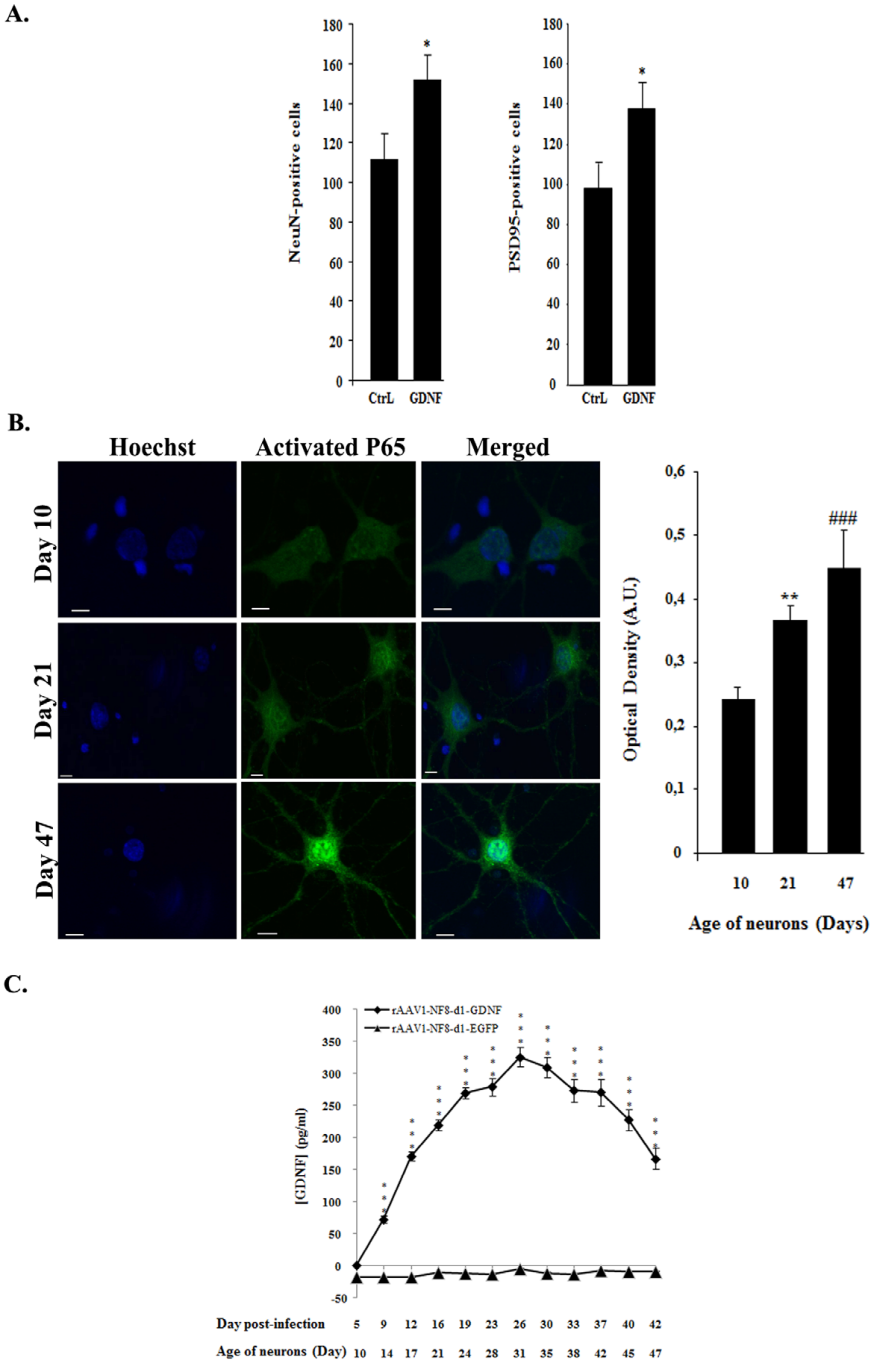
Enhanced neuronal survival in senescent cultures of cortical neurons mediated by rAAV2/1-NF8d1-driven GDNF transgene expression. (**A**) Embryonic day 16 cortical neurons were maintained in 96 wells plates for 60 days. Cultures were fixed and stained with NeuN (a neuronal specific nuclear protein) or PSD95 (a neuronal postsynaptic density protein) a class of anchoring proteins which serve to localize various neuronal ion channels to post-synaptic densities antibodies. The number of NeuN-positive or PSD95-positive neurons is significantly higher in cultures treated with rAAV2/1-NF8d1-GDNF as compared to rAAV2/1-NF8d1-EGFP (CtrL) (p<0.05; student test, n = 10). Bars represent means ± SEM. (**B**) Similar cultures seeded on coated glass coverslips were fixed at day 10, day 21 and day 47 and stained with antibodies directed against activated NFkB followed by a secondary antibody coupled with cyanine 2. Shown are confocal microscopy photographs. Bars represent 50 µM. The mean optical density of the nuclei was measured using the Image J software. Differences between day 10 (n = 39) and day 21 (n = 59) or day 47 (n = 57) were respectively highly significant (**; p<0.001) and very highly significant (###; p<0.0001). The experiment was repeated twice with similar results. (**C**) Culture media from rAAV1-NF8-d1-GDNF- and rAAV1-NF8-d1-EGFP-infected cultures were harvested from day 5 to day 42 post-infection and GDNF concentrations (pg/ml) were measured using ELISA assay. For rAAV1-NF8-d1-GDNF-infected cultures, all values, except at day 5, were significantly higher (***; p<0.0001; n = 10 for rAAV1-NF8-d1-hGDNF and n = 8 for rAAV1-NF8-d1-EGFP) than the basal level (medium from rAAV1-NF8-d1-EGFP-infected cultures). The experiment was repeated twice with similar results.

After 60 days, cells infected with the rAAV2/1-NF8-d1 vectors were fixed and expression of NeuN (a neuronal specific nuclear protein also known as Fox3a) and PSD95 (a neuronal postsynaptic density protein) were evaluated. The number of NeuN-positive and PSD95-positive structures was significantly higher in the rAAV2/1-NF8-d1-GDNF-infected wells compared to rAAV2/1-NF8-EGFP-infected wells (see Fig. 3A). We then tested whether NFκB was activated in aging culture. Parallel cultures on coated glass coverslips in 48 well cultures dishes were fixed at different time points and processed for immunofluorescence using an antibody recognizing the activated form of NFκB. The intensity of the nuclear labeling was quantified and shown to increase with time from day 10 to day 47 after seeding (Fig. 3B).

We further measured the concentrations of secreted GDNF in the culture media. As shown in Figure 3C, the GDNF concentration increased over time until 26 days post-infection (corresponding to 31 days post-seeding), then decreased. The GDNF decrease from 33 days post-infection (38 days post-seeding) was presumably due to a reduced number of neurons in aging cultures which were previously shown to undergo apoptosis (data not shown).

Altogether, these data suggest that GDNF expressed in response to NFκB activation exert a protective effect on aging neurons.

### 3. Selective Induction of rAAV1-NF-mediated Gene Expression in Hippocampal Neurons and Astrocytes in an *in vivo* Model of Epilepsy

In order to show proof of principle of pathology-induced expression from our new gene transfer vector *in vivo*, we tested its activity in a KA-induced rat SE model for temporal lobe epilepsy [16], [36].

The rAAV2/1-NF8-d1 recombinant virus with an EGFP reporter gene (8×10^6^ viral genomes in 2 µl) was injected in the right hippocampus one month prior to SE induction. Eight animals received KA and 8 control animals received an equivalent number of saline injections.

**Figure 4:**
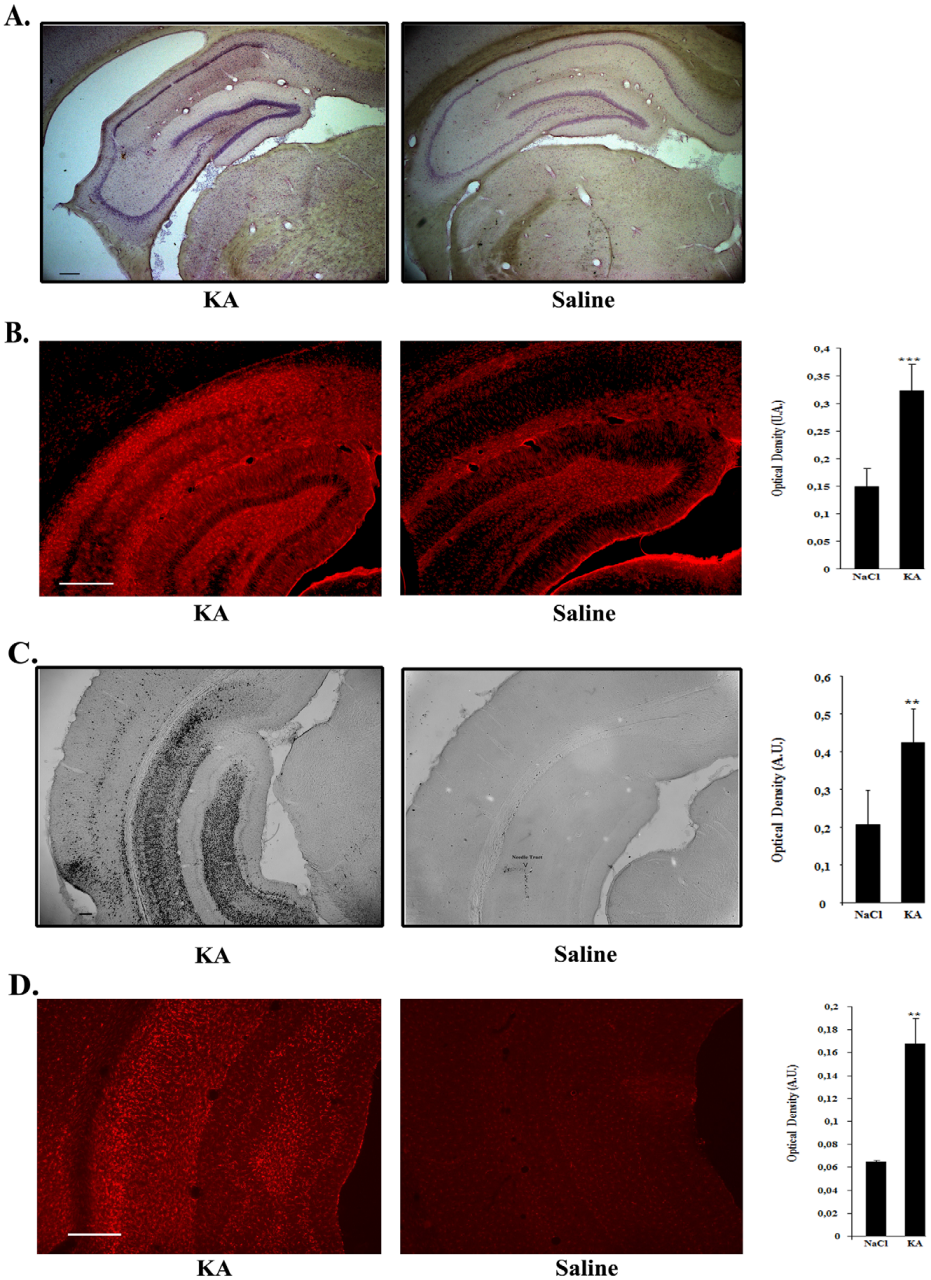
Hippocampal sclerosis and upregulation of inflammatory markers in the hippocampus of kainic acid-treated rats. **A.** Haematoxylin-eosin staining showing typical hippocampal sclerosis in kainic acid-treated rats (KA) (left) as compared to controls (right). Bar represents 200 µm. **B.** Comparison of the intensity of the GFAP astrocytic marker staining in kainic acid- and saline-treated rats. Vibratome brain sections (50 µm) were immunolabeled using mouse anti-GFAP followed by a secondary antibody coupled to Cy3 fluorophore. The intensity of the staining of three random areas in the hipocampal layers on every section was quantified using the Image J program. Data are expressed as the mean optical density ± SEM (n = 8 for each group of animals). The difference between kainic acid and saline-treated groups was highly significant (student test, P = 0,00018). Bar represents 500 µm. **C.** Comparison of the intensity of the CD11b staining of activated microglia in kainic acid- and saline-treated rats. Vibratome brain sections (50 µm) were immunolabeled using mouse anti-CD11b followed by streptavidin-biotin-peroxidase staining. The intensity of the staining of three random areas within the hippocampus on every section was quantified using the Image J program. Arrow shows localized staining at the level of the needle tract. Data are expressed as the mean optical density ± SEM (n = 4 for each group of animals). The difference between kainic acid and saline-treated groups was significant (student test, P = 0,0068). Bar represents 200 µm. **D.** Comparison of the intensity of the IbaI microglial staining in kainic acid- and saline-treated rats. Vibratome brain sections (50 µm) were immunolabeled using goat anti-IbaI followed by anti-goat antibody coupled with cyanine 3. The intensity of the staining of three random areas within the hippocampus on every section was quantified using the Image J program. Data are expressed as the mean optical density ± SEM (n = 6 for each group of animals). The difference between kainic acid and saline-treated groups was significant (student test, p = 0,00121). Bar represents 500 µm.

Haematoxylin-eosin staining of coronal brain sections of KA-treated rats showed typical hippocampal sclerosis whereas hippocampus from saline-treated rats had a normal morphology (Fig. 4A). Activated astrocytes and microglia were evidenced in these regions as demonstrated by GFAP (Fig. 4B) CD11b (Fig. 4C) and IbaI (Fig. 4D) stainings. Quantification of the staining by optical density showed that GFAP, CD11b and IbaI expression was significantly stronger in KA-treated versus saline treated animals (for GFAP: p = 0.0003, n = 8 for each group; for CD11b: p = 0.0135; n = 4 for each group; for IbaI: p = 0.0012; n = 6 for each group).

**Figure 5:**
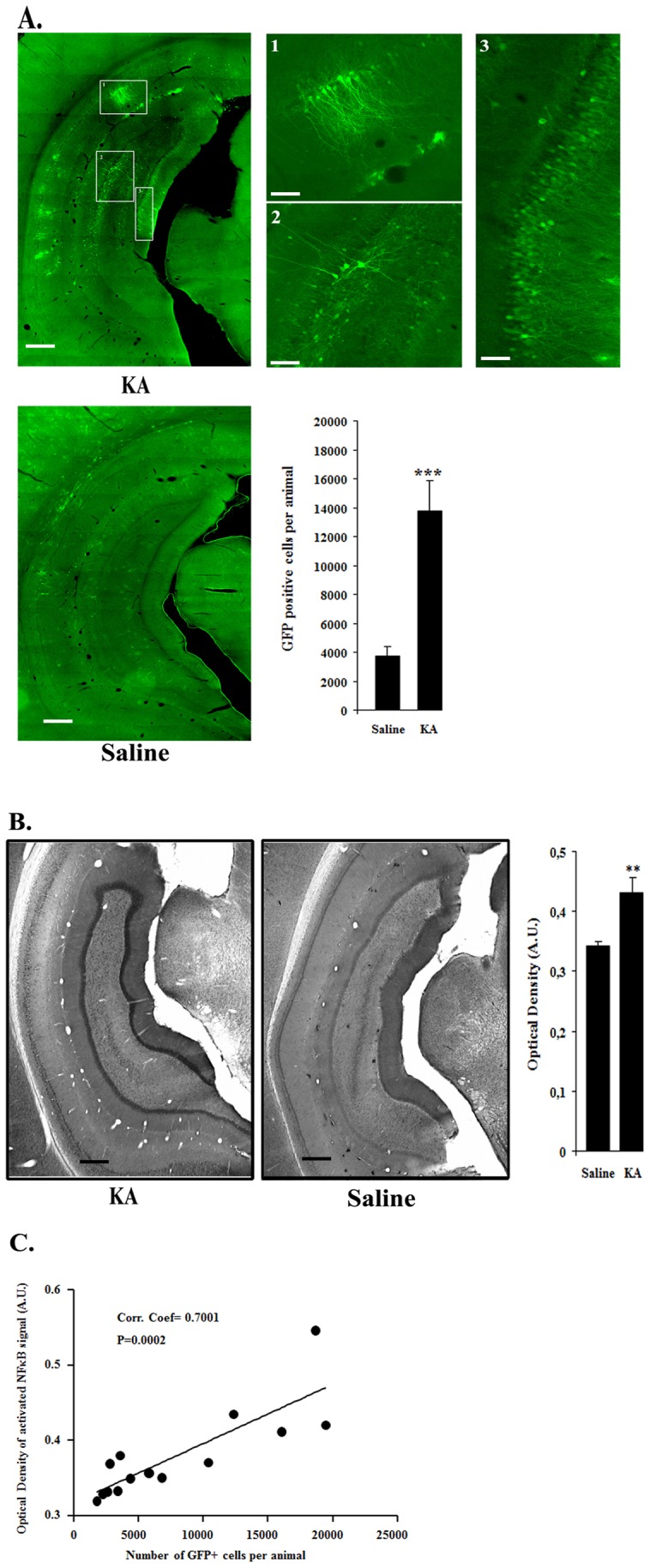
Disease-inducible rAAV1-NF-mediated transgene expression in the hippocampus of kainic acid-treated rats. Recombinant AAV2/1-NF8-d1-EGFP (8×10^6^ vg) was stereotaxically injected in the right hippocampus of male Wistar rats. Animals were kept in two groups: one group was intraperitoneally-injected with kainic acid (n = 8) one month post stereotaxy and the other group received only saline (n = 8). The animals were sacrificed one week after injection. Vibratome brain sections (50 µm) were immunolabeled using an anti-GFP antibody, biotin-streptavidin amplification and Cy2 fluorophore. **A.** Representative examples of GFP-labeling of the CA hippocampal layers dentate gyrus post-kainic acid injection. Note the massive presence of GFP-positive cells in the CA1 and CA3 layers. Stereological counts of GFP-positive cell numbers per animal demonstrates a highly significant induction of the AAV-NF vector by kainic acid (P = 0,000875; student test; n = 8 for the saline group; n = 6 for the kainic acid group). KA, kainic acid; saline, 0.9% NaCl. The experiment was repeated 3 times with similar results. Bars represent 500 µm and 100µm in lower and higher (1,2,3 subpannels) magnification pictures, respectively. **B**. Comparison of the staining intensity for activated NFκB in kainic acid- and saline-treated rats. Vibratome brain sections (50 µm) were immunolabeled using anti-activated NFκB antibody followed by streptavidin-biotin-peroxidase staining. Note, in particular, the localized increased staining in the CA hippocampal layers. The intensity of the staining of three random areas in the hipocampal layers on every section was quantified using the Image J program. Data are expressed as the mean optical density ± SEM (n = 8 for each group of animals). The difference between KA and saline-treated groups was significant (student test, P = 0.0010). Bars represent 500 µm. **C.** Correlation between NFκB activation and GFP expression mediated by rAAV2/1-NF8-d1-EGFP in hippocampal layers. A group of 16 rats was injected with a rAAV1/2-NF8-d1-EGFP in the hippocampus. 8 rats were injected with KA and 8 other animals were injected with the saline solution. Two KA-treated rats were removed from the analysis since they either contained no GFP-positive cells (presumably due to a failure to inject the virus) or had a totally different profile of GFP-expression (no GFP-positive cells in the hippocampal layers, presumably due to wrong stereotaxic coordinates). The total number of GFP-immunoreactive cells per animal (as evaluated by stereology was correlated with the mean optical density of the NFκB stainings in the region containing the transduced cells. There was a significant correlation between the two parameters with a coefficient of 0.7001 (p = 0.0002; n = 6 for KA-treated rats and n = 8 for saline-treated rats). The experiment was repeated a second time (n = 8 for KA-treated rats and n = 7 for saline-treated rats) with similar results.

Prominent gene expression was evidenced in the hippocampus of KA-injected rats whereas only few dispersed GFP-positive cells were detected in saline-injected animals (Fig. 5A). The regional pattern of GFP expression in the SE animals consisted of a preferential transduction of the CA1 and CA3 layers as well as an occasional labeling of cells in the stratum oriens (see Fig. 5A). GFP-positive cells were present in a region of 2.5 mm along the antero-posterior axis (data not shown). Among the eight KA-treated rats, one had no GFP-positive cell and in another rat the biodistribution of gfp-positive cells was totally different (the labeling was observed only in stratum orens and absent in the hippocampal layers). It was considered that stereotaxic injections of the virus were performed at slightly different coordinates in these 2 animals and they were withdrawn for further analysis.

In order to correlate GFP expression with NFκB activation, hippocampal sections were also labeled with antibodies directed against activated NFκB. These results show a strong staining in specific hippocampal subregions of epileptic rats corresponding to the area in which GFP-positive cells were observed (see Fig. 5B).

The number of GFP-positive cells was also evaluated by stereology and compared to the control group. The data showed that the epileptic animals contained significantly more GFP-positive cells than the control group (Fig. 5A). Furthermore a significant correlation between the GFP positive cell numbers and activated NFκB staining intensity per animal was observed with a coefficient of 0.7001, (p = 0.0002) (Fig. 5C).

**Figure 6:**
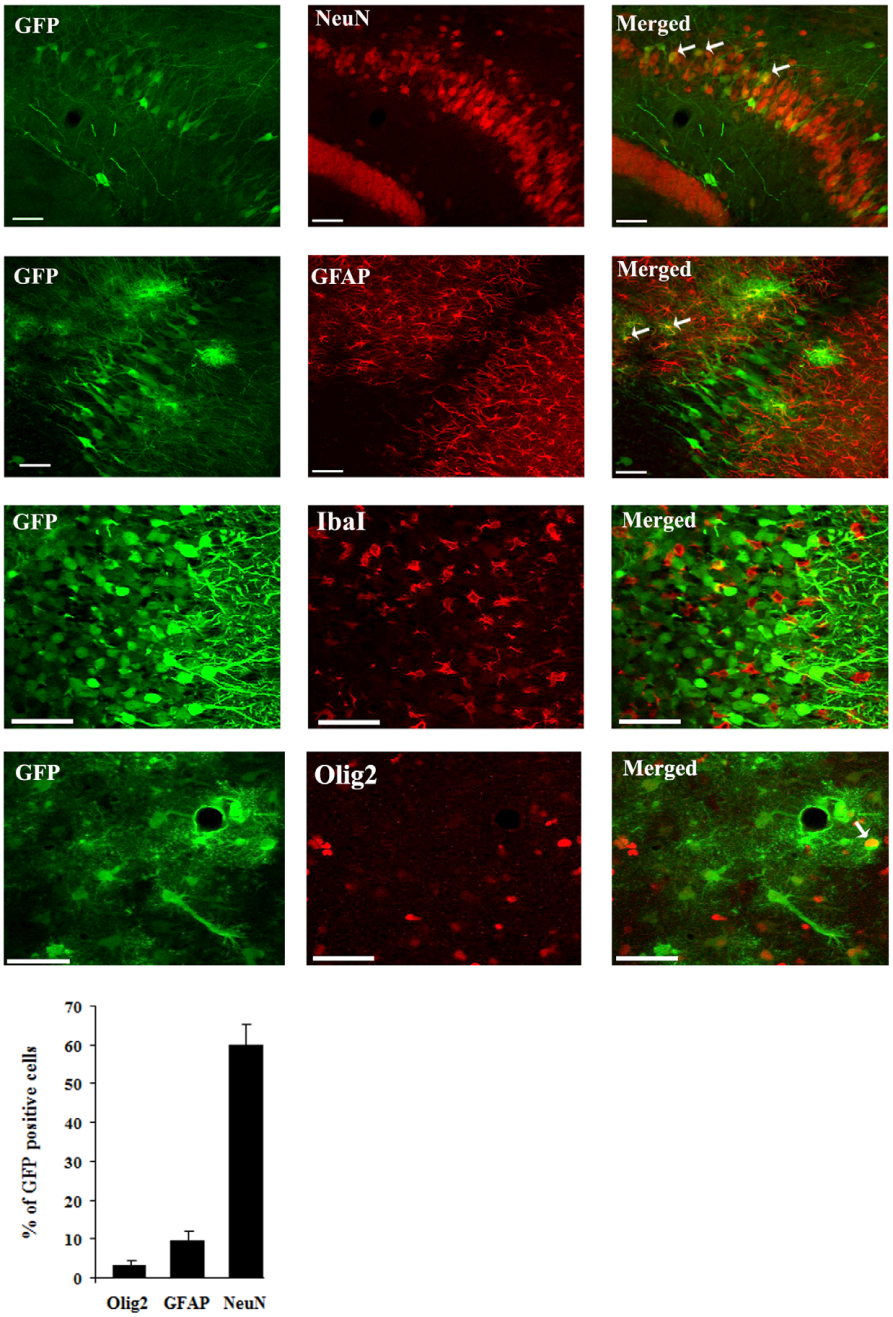
Cellular specificity of rAAV2/1-NF8d1-EGFP-mediated gene transfer in the hippocampus of kainic acid-treated rats. Kainic acid (5 mg/kg) was injected intraperitoneally (ip) one month after stereotaxic injection of rAAV2/1-NF8d1-EGFP into the hippocampus. Fifty µm coronal sections were co-labeled with GFP (green fluorescence) and NeuN, GFAP IbaI or Olig2. Confocal pictures showing GFP and cell-specific markers co-labeled cells (yellow). White arrows indicate co-labeled cells. Bars represent 50 µm. The mean percentage of GFP-positive cells co-labeling with the GFAP, NeuN and Olig2 markers are shown. No GFP-positive cell stained positive for IbaI. Analysis was performed using confocal microscopy and counting the number co-labeled cells on 5 sections per animal (n = 5 rats for GFP/NeuN and GFP/GFAP, n = 6 for GFP/IbaI and n = 7 for GFP/Olig2 co-labeling).

The majority of GFP-positive cells (60%) were neurons (as revealed by double GFP-NeuN immunofluorescence and confocal microscopy), with a smaller but nonetheless statistically significant proportion (9.5%) of transduced cells comprising astrocytes (based on co-labeling for the astrocytic marker GFAP) as well as 3.2% of cells of the oligodendrocyte lineage (as revealed using the Olig2 marker [47] (Fig. 6). In contrast, double immunofluorescence for GFP and the IbaI marker for microglia revealed no co-labeled cell (Fig. 6).

The experiment was repeated a second time (n = 8 for the KA group and n = 7 for the saline group) and similar results were obtained (data not shown).

**Figure 7:**
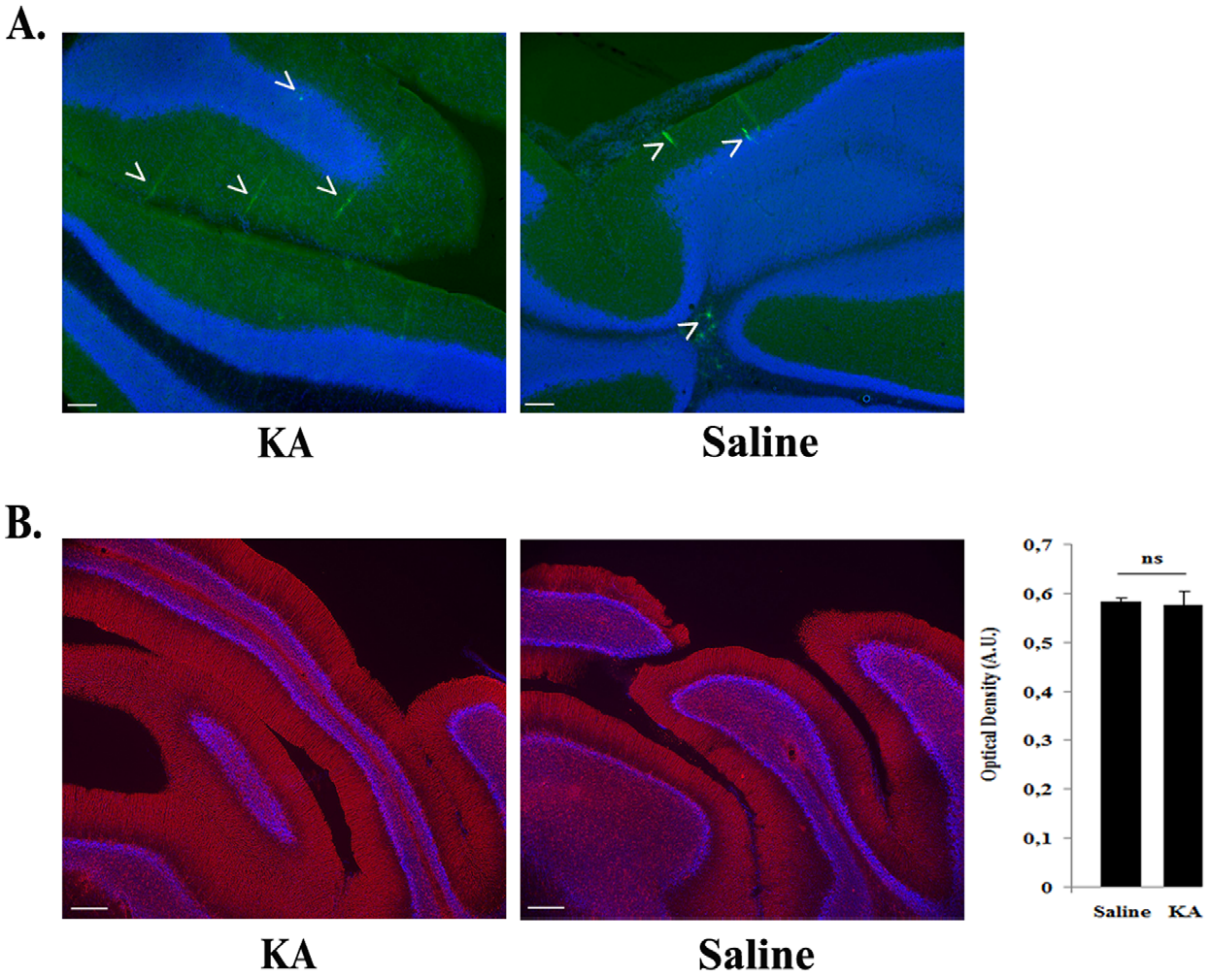
Absence of transgene upregulation in rAAV2/1-NF8d1-EGFP-mediated gene transfer in the cerebellum of kainic acid-treated rats. Kainic acid (5 mg/kg) was injected intraperitoneally (ip) one month after stereotaxic injection of rAAV2/1-NF8d1-EGFP into the cerebellum. Fifty µm coronal sections were labeled with GFP or GFAP antibodies. Confocal pictures showing GFP (A) or GFAP (B). Bars represent 50 µm. GFAP staining was quantified by measuring the optical density of three area in all sections stained per animal (6 rats for each condition). The difference between KA- and saline-treated rats is not significant (student test, p = 0,8612) (B).

In order to further demonstrate that vector induction was dependent on the KA-induced NFκB activation, the cerebellum was selected as negative control vector injection site because lack of NFκB activation following kainic acid administration to adult rats was previously shown in this region [48]. As expected, i) GFP-positive cells were restricted in a small area around the injection site and their number was not increased by KA treatment (Fig. 7A); ii) KA did not induce cerebellar inflammation as suggested by the similar GFAP staining in treated and untreated rats (Fig. 7B).

## Discussion

Abnormal activation of NFκB is a heavily implicated pathogenic mechanism in a number of chronic neurodegenerative and neuroinflammatory diseases [49]. In the CNS, neuroinflammation propagates by communication between astrocytes, microglia and neurons through the activity of pro-inflammatory cytokines such as TNFα and IL1βwhich activate NFκB [50], [51]. While neurons require a basal level of activated NFκB for their survival [25], uncontrolled neuronal activation results in excitotoxicity which leads to further NFκB activation [52]. Our goal was to design a gene transfer vector able to sense pathological increases of NFκB activation.

In order to design a tight NFκB -responsive AAV vector, we first selected a promoter sequence on the basis of previous studies with tetracycline-inducible vectors. Strathdee and colleagues [43] have previously compared the minimal thymidine kinase promoter (mTK) and CMV promoter (mCMV) and reported that mTK conferred a tighter control to a tetracycline-responsive promoter. Unexpectedly, however, our test of 4 copies of the JC virus NFκB-responsive element linked to the same two promoters showed that the AAV-mTK-NF4 vector conveyed a high basal level of expression and was poorly (less than 2-fold) inducible by TNFα in HEK-293T cells. We have not explicitly tested the origin of this high basal expression level, which could reflect activity of the mTK promoter alone or together with a contribution from incompletely blocked ITR enhancer activity.

To overcome the issues of high basal expression and poor inducibility, we constructed and tested a new variant of a minimal CMV promoter (mCMVΔ2). It is shown that mCMVΔ2 conferred to AAV-NF a lower basal transgene expression level than the mTK promoter while exhibiting a higher (approx. 4-fold) inducibility in response to TNFα.

To further reduce the basal level of transcription from mCMVΔ2, we modified the distance between the viral *cis-acting* elements (left ITR) and NFκB-RE and the TATA box of the promoter. Increasing the distance to 31 (d1) and 71 bp (d3) resulted in a further decrease (5- and 13- fold, respectively) of the basal expression level. To increase the inducibility of the vector, we have increased the number of NFκB-REs. The constructs harbouring 8 REs (pSC-NF8-d1 and pSC-NF8-d3) had a higher inducibility than those harbouring 4 REs (pSC-NF4-d1 and pSC-NF4-d3) while retaining a similar background (for pSC-NF8-d3, data not shown). This suggests that the basal expression of the vector may depend more on the ITR than the NFκB-REs. Increasing the number of NFκB-REs to 12 did not further increase the inducibility but slightly reduced the basal level (compare pSC-NF8-d1-EGFP versus pSC-NF12-d1-EGFP), possibly due to an increase of the distance from the minimal promoter to the left ITR.

Two models in which NFκB activation occurs were used to prove the responsiveness of the optimized NFκB -inducible AAV vector. These consisted of an *in vitro* model of neuronal aging and a rat model of temporal lobe epilepsy.

NFκB involvement in the former is believed to result from increases in inflammatory cytokines with age [20] and Alzheimer Disease [21]. Additional data suggest that NFκB activation is not an autocompensatory response, since NFκB activation fails to protect neurons again apoptosis associated with long-term culturing or dual TNFα and amyloid-beta toxicity [22].

GDNF has previously been shown to be neuroprotective in animal models of brain aging [53] as well as to reduce apoptosis in dopaminergic neurons [54]. In the current study, we show that GDNF expression under the control of our NFκB-inducible vector increased (by 40%) the number of aging cortical neurons in culture. Our data suggest that it is feasible to harness age-related NFκB activation to express neuroprotective factors in age-related diseases.

A chronic inflammatory state in the brain has been associated with epilepsy in both human and rodent studies [55]–[58]. In addition, proinflammatory molecules exacerbate seizures in experimental models, whereas anti-inflammatory drugs can have anticonvulsant efficacy [59]. The observed reporter gene expression mediated by the NFκB-inducible AAV vector in the hippocampus in response to systemic KA injection is in accordance with these observations, and further suggests that NFκB activation could be harnessed to drive expression of anti-inflammatory genes. In addition, the cell-type specificity of GFP expression might provide clues to the mechanisms of neuroinflammation in induction and progression of epileptic condition. As expected, in the cerebellum, in which NFκB is not induced by intraperitoneal injection of KA [48] AAV-NF-mediated GFP expression remained at the basal level in epileptic rats.

Transgene expression was detected in neurons and cells of the oligodendrocyte lineage showing that vector delivery and NFκB activation occur in these 3 cell types in the KA model. NFκB activation in neurons could reflect secondary neuroinflammation in neurons by pro-inflammatory cytokines released by microglia and/or neuron hyperactivity induced by the kainic acid model itself [60].

In contrast, no GFP expression was evidenced in microglial cells. This is surprising, since a NFκB response has been described in microglia activated by the pro-inflammatory cytokine TNFα [61] and KA treatment is known to induce pro-inflammatory cytokines, including TNFα in the hippocampus [47].

The capsid serotype largely influences the cellular tropism of AAV vectors in the CNS [62]–[65]. In rodents, the majority of AAV serotypes or capsid variants, when injected in the rat parenchyma mediate transgene expression mostly in neurons with notable exceptions such as rAAV9 which mediates transgene expression in both neurons and astrocytes (Foust et al., 2009) or rAAV4 which transduce ependymal cells [66]. Interestingly, enhanced gene delivery into astrocytes has been obtained by selecting AAV capsid variants through molecular evolution [67].

In addition, we and others have previously shown that, the promoter also influences the AAV-delivered transgene expression profile [68], [69]. Indeed, using a serotype 1 capsid, GFP was exclusively expressed in neurons using the tetOn promoter whereas 5% of GFP-positive cells were astrocytes with the CMV promoter. In the present study, with the rAAV1-NF8-d1 vector, we observed that 9.5% of GFP-positive cells in the hippocampal CA1 and CA3 layers of epileptic rats were astrocytes whereas 60% were neurons. Whether these data reflect the proportion of astrocytes and neurons in which NFκB has been activated by the KA treatment or are biased by the tropism of the AAV serotype 1 capsid and mCMV promoter cannot be concluded from our data.

It remains to be determined if the use of other AAV serotypes would modify the proportion of neurons, astrocytes and possibly microglial cells expressing a transgene driven by the AAV-NF vector. Furthermore, the optimal targeting of glial versus neuronal cells may also vary by disease indication.

The onset of AAV-mediated transgene expression in the brain is fairly slow [38]. However, serotype 1 AAV vectors are characterized by remarkably rapid kinetics in several regions of the brain with maximal expression reached at 2 to 4 days post-injection [70]; [71]. In addition, self-complementary vectors that bypass the limitation of second-strand viral DNA synthesis further reduce the delay for transgene expression [70]; [29].

In conclusion, this study constitutes a proof-of-concept step toward the use of NFκB-inducible AAV vectors for disease-inducible transgene delivery to treat disorders of the nervous system. Nonetheless, more data will be required to establish whether transgene expression tightly follows the temporal course of disease and whether pathology-appropriate cellular targeting of the inflammatory response can be reliably achieved.

## References

[1] Fluri DA, Baba MD, Fussenegger M (2007) Adeno-associated viral vectors engineered for macrolide-adjustable transgene expression in mammalian cells and mice. BMC Biotechnol 7: 75. 17986332 10.1186/1472-6750-7-75 PMC2211474

[2] Gossen M, Freundlieb S, Bender G, Muller G, Hillen W, et al (1995) Transcriptional activation by tetracyclines in mammalian cells. Science 268: 1766–1769. 7792603 10.1126/science.7792603

[3] Adriaansen J, Khoury M, de Cortie CJ, Fallaux FJ, Bigey P, et al (2007) Reduction of arthritis following intra-articular administration of an adeno-associated virus serotype 5 expressing a disease-inducible TNF-blocking agent. Ann Rheum Dis 66: 1143–1150. 17363402 10.1136/ard.2006.064519 PMC1955149

[4] Jakobsson J, Georgievska B, Ericson C, Lundberg C (2004) Lesion-dependent regulation of transgene expression in the rat brain using a human glial fibrillary acidic protein-lentiviral vector. Eur J Neurosci 19: 761–765. 14984426 10.1111/j.0953-816x.2003.03147.x

[5] Phillips MI, Tang Y, Schmidt-Ott K, Qian K, Kagiyama S (2002) Vigilant vector: heart-specific promoter in an adeno-associated virus vector for cardioprotection. Hypertension 39: 651–655. 11882625 10.1161/hy0202.103472

[6] Siebenlist U, Franzoso G, Brown K (1994) Structure, regulation and function of NF-kappa B. Annu Rev Cell Biol. 10: 405–455. 10.1146/annurev.cb.10.110194.002201 7888182

[7] Karin M, Delhase M (2000) The I kappa B kinase (IKK) and NF-kappa B: key elements of proinflammatory signalling. Semin Immunol 12: 85–98. 10723801 10.1006/smim.2000.0210

[8] Kaltschmidt B, Widera D, Kaltschmidt C (2005) Signaling via NF-kappaB in the nervous system. Biochim Biophys Acta 1745: 287–299. 15993497 10.1016/j.bbamcr.2005.05.009

[9] Lubin FD, Johnston LD, Sweatt JD, Anderson AE (2005) Kainate mediates nuclear factor-kappa B activation in hippocampus via phosphatidylinositol-3 kinase and extracellular signal-regulated protein kinase. Neuroscience 133: 969–981. 15916859 10.1016/j.neuroscience.2005.03.028

[10] Lubin FD, Ren Y, Xu X, Anderson AE (2007) Nuclear factor-kappa B regulates seizure threshold and gene transcription following convulsant stimulation. J Neurochem 103: 1381–1395. 17727632 10.1111/j.1471-4159.2007.04863.x

[11] Ben Ari Y (1985) Limbic seizure and brain damage produced by kainic acid: mechanisms and relevance to human temporal lobe epilepsy. Neuroscience 14: 375–403. 2859548 10.1016/0306-4522(85)90299-4

[12] Cavalheiro EA, Leite JP, Bortolotto ZA, Turski WA, Ikonomidou C, et al (1991) Long-term effects of pilocarpine in rats: structural damage of the brain triggers kindling and spontaneous recurrent seizures. Epilepsia 32: 778–782. 1743148 10.1111/j.1528-1157.1991.tb05533.x

[13] Lothman EW, Bertram EH, Kapur J, Stringer JL (1990) Recurrent spontaneous hippocampal seizures in the rat as a chronic sequela to limbic status epilepticus. Epilepsy Res 6: 110–118. 2387285 10.1016/0920-1211(90)90085-a

[14] Vincent P, Mulle C (2009) Kainate receptors in epilepsy and excitotoxicity. Neuroscience 158: 309–323. 18400404 10.1016/j.neuroscience.2008.02.066

[15] Bouilleret V, Ridoux V, Depaulis A, Marescaux C, Nehlig A, et al (1999) Recurrent seizures and hippocampal sclerosis following intrahippocampal kainate injection in adult mice: electroencephalography, histopathology and synaptic reorganization similar to mesial temporal lobe epilepsy. Neuroscience 89: 717–729. 10199607 10.1016/s0306-4522(98)00401-1

[16] Hellier JL, Patrylo PR, Buckmaster PS, Dudek FE (1998) Recurrent spontaneous motor seizures after repeated low-dose systemic treatment with kainate: assessment of a rat model of temporal lobe epilepsy. Epilepsy Res 31: 73–84. 9696302 10.1016/s0920-1211(98)00017-5

[17] Majores M, Eils J, Wiestler OD, Becker AJ (2004) Molecular profiling of temporal lobe epilepsy: comparison of data from human tissue samples and animal models. Epilepsy Res 60: 173–178. 15380561 10.1016/j.eplepsyres.2004.07.002

[18] Ravizza T, Balosso S, Vezzani A (2011) Inflammation and prevention of epileptogenesis. Neurosci Lett 497: 223–230. 21362451 10.1016/j.neulet.2011.02.040

[19] Stafstrom CE, Thompson JL, Holmes GL (1992) Kainic acid seizures in the developing brain: status epilepticus and spontaneous recurrent seizures. Brain Res Dev Brain Res 65: 227–236. 1572066 10.1016/0165-3806(92)90184-x

[20] Gemma C, Mesches MH, Sepesi B, Choo K, Holmes DB, et al (2002) Diets enriched in foods with high antioxidant activity reverse age-induced decreases in cerebellar beta-adrenergic function and increases in proinflammatory cytokines. J Neurosci 22: 6114–6120. 12122072 10.1523/JNEUROSCI.22-14-06114.2002 PMC6757915

[21] Tarkowski E, Liljeroth AM, Minthon L, Tarkowski A, Wallin A, et al (2003) Cerebral pattern of pro- and anti-inflammatory cytokines in dementias. Brain Res Bull 61: 255–260. 12909295 10.1016/s0361-9230(03)00088-1

[22] Patel JR, Brewer GJ (2008) Age-related differences in NFkappaB translocation and Bcl-2/Bax ratio caused by TNFalpha and Abeta42 promote survival in middle-age neurons and death in old neurons. Exp Neurol 213: 93–100. 18625500 10.1016/j.expneurol.2008.05.007 PMC2597588

[23] Mattson MP, Meffert MK (2006) Roles for NF-kappaB in nerve cell survival, plasticity, and disease. Cell Death Differ 13: 852–860. 16397579 10.1038/sj.cdd.4401837

[24] Carlsen H, Moskaug JO, Fromm SH, Blomhoff R (2002) In vivo imaging of NF-kappa B activity. J Immunol 168: 1441–1446. 11801687 10.4049/jimmunol.168.3.1441

[25] Bhakar AL, Tannis LL, Zeindler C, Russo MP, Jobin C, et al (2002) Constitutive nuclear factor-kappa B activity is required for central neuron survival. J Neurosci 22: 8466–8475. 12351721 10.1523/JNEUROSCI.22-19-08466.2002 PMC6757785

[26] Ranganathan PN, Khalili K (1993) The transcriptional enhancer element, kappa B, regulates promoter activity of the human neurotropic virus, JCV, in cells derived from the CNS. Nucleic Acids Res 21: 1959–1964. 8388103 10.1093/nar/21.8.1959 PMC309438

[27] Wollebo HS, Safak M, Del VL, Khalili K, White MK (2011) Role for tumor necrosis factor-alpha in JC virus reactivation and progressive multifocal leukoencephalopathy. J Neuroimmunol 233: 46–53. 21185609 10.1016/j.jneuroim.2010.11.013 PMC3074035

[28] Lesuisse C, Martin LJ (2002) Immature and mature cortical neurons engage different apoptotic mechanisms involving caspase-3 and the mitogen-activated protein kinase pathway. J Cereb Blood Flow Metab 22: 935–950. 12172379 10.1097/00004647-200208000-00005

[29] McCarty DM, Fu H, Monahan PE, Toulson CE, Naik P, et al (2003) Adeno-associated virus terminal repeat (TR) mutant generates self-complementary vectors to overcome the rate-limiting step to transduction in vivo. Gene Ther 10: 2112–2118. 14625565 10.1038/sj.gt.3302134

[30] Zolotukhin S, Byrne BJ, Mason E, Zolotukhin I, Potter M, et al (1999) Recombinant adeno-associated virus purification using novel methods improves infectious titer and yield. Gene Ther 6: 973–985. 10455399 10.1038/sj.gt.3300938

[31] Lock M, McGorray S, Auricchio A, Ayuso E, Beecham EJ, et al (2010) Characterization of a recombinant adeno-associated virus type 2 Reference Standard Material. Hum Gene Ther 21: 1273–1285. 20486768 10.1089/hum.2009.223 PMC2957240

[32] Zala D, Benchoua A, Brouillet E, Perrin V, Gaillard MC, et al (2005) Progressive and selective striatal degeneration in primary neuronal cultures using lentiviral vector coding for a mutant huntingtin fragment. Neurobiol Dis 20: 785–798. 16006135 10.1016/j.nbd.2005.05.017

[33] Zabel U, Henkel T, Silva MS, Baeuerle PA (1993) Nuclear uptake control of NF-kappa B by MAD-3, an I kappa B protein present in the nucleus. EMBO J 12: 201–211. 7679069 10.1002/j.1460-2075.1993.tb05646.x PMC413192

[34] Vermoesen K, Smolders I, Massie A, Michotte Y, Clinckers R (2010) The control of kainic acid-induced status epilepticus. Epilepsy Res 90: 164–166. 20434312 10.1016/j.eplepsyres.2010.04.001

[35] Vermoesen K, Massie A, Smolders I, Clinckers R (2012) The antidepressants citalopram and reboxetine reduce seizure frequency in rats with chronic epilepsy. Epilepsia 53: 870–878. 10.1111/j.1528-1167.2012.03436.x [doi]. 10.1111/j.1528-1167.2012.03436.x 22429158

[36] Williams PA, White AM, Clark S, Ferraro DJ, Swiercz W, et al (2009) Development of spontaneous recurrent seizures after kainate-induced status epilepticus. J Neurosci 29: 2103–2112. 19228963 10.1523/JNEUROSCI.0980-08.2009 PMC2897752

[37] Fu H, Muenzer J, Samulski RJ, Breese G, Sifford J, et al (2003) Self-complementary adeno-associated virus serotype 2 vector: global distribution and broad dispersion of AAV-mediated transgene expression in mouse brain. Mol Ther 8: 911–917. 14664793 10.1016/j.ymthe.2003.08.021

[38] McCarty DM, Monahan PE, Samulski RJ (2001) Self-complementary recombinant adeno-associated virus (scAAV) vectors promote efficient transduction independently of DNA synthesis. Gene Ther 8: 1248–1254. 11509958 10.1038/sj.gt.3301514

[39] Afione SA, Conrad CK, Kearns WG, Chunduru S, Adams R, et al (1996) In vivo model of adeno-associated virus vector persistence and rescue. J Virol 70: 3235–3241. 8627804 10.1128/jvi.70.5.3235-3241.1996 PMC190187

[40] Haberman RP, McCown TJ, Samulski RJ (2000) Novel transcriptional regulatory signals in the adeno-associated virus terminal repeat A/D junction element. J Virol 74: 8732–8739. 10954575 10.1128/jvi.74.18.8732-8739.2000 PMC116385

[41] Eggermont J, Proudfoot NJ (1993) Poly(A) signals and transcriptional pause sites combine to prevent interference between RNA polymerase II promoters. EMBO J 12: 2539–2548. 8508777 10.1002/j.1460-2075.1993.tb05909.x PMC413492

[42] Chtarto A, Bender HU, Hanemann CO, Kemp T, Lehtonen E, et al (2003) Tetracycline-inducible transgene expression mediated by a single AAV vector. Gene Ther 10: 84–94. 12525840 10.1038/sj.gt.3301838

[43] Strathdee CA, McLeod MR, Hall JR (1999) Efficient control of tetracycline-responsive gene expression from an autoregulated bi-directional expression vector. Gene 229: 21–29. 10095100 10.1016/s0378-1119(99)00045-1

[44] Channavajhala PL, Rao VR, Spaulding V, Lin LL, Zhang YG (2005) hKSR-2 inhibits MEKK3-activated MAP kinase and NF-kappaB pathways in inflammation. Biochem Biophys Res Commun 334: 1214–1218. 16039990 10.1016/j.bbrc.2005.07.009

[45] Boshart M, Weber F, Jahn G, Dorsch-Hasler K, Fleckenstein B, et al (1985) A very strong enhancer is located upstream of an immediate early gene of human cytomegalovirus. Cell 41: 521–530. 2985280 10.1016/s0092-8674(85)80025-8

[46] Howard DB, Powers K, Wang Y, Harvey BK (2008) Tropism and toxicity of adeno-associated viral vector serotypes 1, 2, 5, 6, 7, 8, and 9 in rat neurons and glia in vitro. Virology 372: 24–34. S0042-6822(07)00658-7 [pii];10.1016/j.virol.2007.10.007 [doi]. 10.1016/j.virol.2007.10.007 PMC2293646 18035387

[47] Somera-Molina KC, Nair S, Van Eldik LJ, Watterson DM, Wainwright MS (2009) Enhanced microglial activation and proinflammatory cytokine upregulation are linked to increased susceptibility to seizures and neurologic injury in a ‘two-hit’ seizure model. Brain Res 1282: 162–172. S0006-8993(09)01093-2 [pii];10.1016/j.brainres.2009.05.073 [doi]. 10.1016/j.brainres.2009.05.073 PMC2739829 19501063

[48] Rong Y, Baudry M (1996) Seizure activity results in a rapid induction of nuclear factor-kappa B in adult but not juvenile rat limbic structures. J Neurochem 67: 662–668. 8764593 10.1046/j.1471-4159.1996.67020662.x

[49] Verma IM (2004) Nuclear factor (NF)-kappaB proteins: therapeutic targets. Ann Rheum Dis 63 Suppl 2: ii57–ii61. 15479873 10.1136/ard.2004.028266 PMC1766777

[50] Depino AM, Earl C, Kaczmarczyk E, Ferrari C, Besedovsky H, et al (2003) Microglial activation with atypical proinflammatory cytokine expression in a rat model of Parkinson’s disease. Eur J Neurosci 18: 2731–2742. 14656322 10.1111/j.1460-9568.2003.03014.x

[51] Tansey MG, Goldberg MS (2010) Neuroinflammation in Parkinson’s disease: its role in neuronal death and implications for therapeutic intervention. Neurobiol Dis 37: 510–518. 19913097 10.1016/j.nbd.2009.11.004 PMC2823829

[52] Caba E, Bahr BA (2004) Biphasic NF-kappaB activation in the excitotoxic hippocampus. Acta Neuropathol 108: 173–182. 15138780 10.1007/s00401-004-0876-5

[53] Emborg ME, Moirano J, Raschke J, Bondarenko V, Zufferey R, et al (2009) Response of aged parkinsonian monkeys to in vivo gene transfer of GDNF. Neurobiol Dis 36: 303–311. 19660547 10.1016/j.nbd.2009.07.022 PMC2989601

[54] Lin LF, Doherty DH, Lile JD, Bektesh S, Collins F (1993) GDNF: a glial cell line-derived neurotrophic factor for midbrain dopaminergic neurons. Science 260: 1130–1132. 8493557 10.1126/science.8493557

[55] Jankowsky JL, Derrick BE, Patterson PH (2000) Cytokine responses to LTP induction in the rat hippocampus: a comparison of in vitro and in vivo techniques. Learn Mem 7: 400–412. 11112799 10.1101/lm.32600 PMC311345

[56] Oprica M, Eriksson C, Schultzberg M (2003) Inflammatory mechanisms associated with brain damage induced by kainic acid with special reference to the interleukin-1 system. J Cell Mol Med 7: 127–140. 12927051 10.1111/j.1582-4934.2003.tb00211.x PMC6740282

[57] Turrin NP, Rivest S (2004) Innate immune reaction in response to seizures: implications for the neuropathology associated with epilepsy. Neurobiol Dis 16: 321–334. 15193289 10.1016/j.nbd.2004.03.010

[58] Vezzani A, Granata T (2005) Brain inflammation in epilepsy: experimental and clinical evidence. Epilepsia 46: 1724–1743. 16302852 10.1111/j.1528-1167.2005.00298.x

[59] Vezzani A, Aronica E, Mazarati A, Pittman QJ (2011) Epilepsy and brain inflammation. Exp Neurol. 10.1016/j.expneurol.2011.09.033 21985866

[60] Lacaille JC, Mueller AL, Kunkel DD, Schwartzkroin PA (1987) Local circuit interactions between oriens/alveus interneurons and CA1 pyramidal cells in hippocampal slices: electrophysiology and morphology. J Neurosci 7: 1979–1993. 3612227 10.1523/JNEUROSCI.07-07-01979.1987 PMC6568928

[61] Sheppard PW, Sun X, Emery JF, Giffard RG, Khammash M (2011) Quantitative characterization and analysis of the dynamic NF-kappaB response in microglia. BMC Bioinformatics 12: 276. 1471–2105–12–276 [pii];10.1186/1471-2105-12-276 [doi]. 10.1186/1471-2105-12-276 PMC3158563 21729324

[62] Blits B, Derks S, Twisk J, Ehlert E, Prins J, et al (2010) Adeno-associated viral vector (AAV)-mediated gene transfer in the red nucleus of the adult rat brain: comparative analysis of the transduction properties of seven AAV serotypes and lentiviral vectors. J Neurosci Methods 185: 257–263. 19850079 10.1016/j.jneumeth.2009.10.009

[63] Burger C, Gorbatyuk OS, Velardo MJ, Peden CS, Williams P, et al (2004) Recombinant AAV viral vectors pseudotyped with viral capsids from serotypes 1, 2, and 5 display differential efficiency and cell tropism after delivery to different regions of the central nervous system. Mol Ther 10: 302–317. 15294177 10.1016/j.ymthe.2004.05.024

[64] Taymans JM, Vandenberghe LH, Haute CV, Thiry I, Deroose CM, et al (2007) Comparative analysis of adeno-associated viral vector serotypes 1, 2, 5, 7, and 8 in mouse brain. Hum Gene Ther 18: 195–206. 17343566 10.1089/hum.2006.178

[65] Van der PA, Toelen J, Carlon M, Van den HC, Coun F, et al (2011) Efficient and stable transduction of dopaminergic neurons in rat substantia nigra by rAAV 2/1, 2/2, 2/5, 2/6.2, 2/7, 2/8 and 2/9. Gene Ther 18: 517–527. 21326331 10.1038/gt.2010.179

[66] Davidson BL, Stein CS, Heth JA, Martins I, Kotin RM, et al. (2000) Recombinant adeno-associated virus type 2, 4, and 5 vectors: transduction of variant cell types and regions in the mammalian central nervous system. Proc Natl Acad Sci U S A 97: 3428–3432. 10.1073/pnas.050581197 [doi];050581197 [pii]. 10.1073/pnas.050581197 PMC16256 10688913

[67] Koerber JT, Klimczak R, Jang JH, Dalkara D, Flannery JG, et al. (2009) Molecular evolution of adeno-associated virus for enhanced glial gene delivery. Mol Ther 17: 2088–2095. mt2009184 [pii];10.1038/mt.2009.184 [doi]. 10.1038/mt.2009.184 PMC2788045 19672246

[68] Bockstael O, Chtarto A, Wakkinen J, Yang X, Melas C, et al. (2008) Differential transgene expression profiles from rAAV2/1 vectors using the tetON and CMV promoters in the rat brain. Hum Gene Ther. 10.1089/hum.2008.099 19866492

[69] Haberman R, Criswell H, Snowdy S, Ming Z, Breese G, et al (2002) Therapeutic liabilities of in vivo viral vector tropism: adeno-associated virus vectors, NMDAR1 antisense, and focal seizure sensitivity. Mol Ther 6: 495–500. 12377191 10.1006/mthe.2002.0701 PMC3213639

[70] Bockstael O, Melas C, Pythoud C, Levivier M, McCarty D, et al. (2012) Rapid Transgene Expression in Multiple Precursor Cell Types of Adult Rat Subventricular Zone Mediated by Adeno-Associated Type 1 Vectors. Hum Gene Ther. 10.1089/hum.2011.216 [doi]. 10.1089/hum.2011.216 PMC3404419 22471423

[71] Reimsnider S, Manfredsson FP, Muzyczka N, Mandel RJ (2007) Time course of transgene expression after intrastriatal pseudotyped rAAV2/1, rAAV2/2, rAAV2/5, and rAAV2/8 transduction in the rat. Mol Ther 15: 1504–1511. 17565350 10.1038/sj.mt.6300227

[72] Zolotukhin S, Potter M, Hauswirth WW, Guy J, Muzyczka N (1996) A “humanized” green fluorescent protein cDNA adapted for high-level expression in mammalian cells. J Virol 70: 4646–4654. 8676491 10.1128/jvi.70.7.4646-4654.1996 PMC190401

